# L-Lactic Acid-Enriched Wheat Bran *Qu* Containing *Bacillus cereus* Regulates L-Lactic Acid and Ester Formation in Light-Flavor Baijiu Fermentation

**DOI:** 10.3390/foods15132290

**Published:** 2026-06-26

**Authors:** Zhiguo Huang, Lvchang Liu, Jin Hua, Yi Wang, Yabin Zhou

**Affiliations:** 1 School of Food and Liquor Engineering, Sichuan University of Science and Engineering (SUSE), Yibin 644000, China; 2 Brewing Science and Technology Key Laboratory of Sichuan Province, Sichuan University of Science and Engineering (SUSE), Yibin 644000, China; 3 College of Public Health and Medicine, Flinders University, Adelaide 5042, Australia; 4 Key Laboratory of Baijiu Supervision Technology, State Administration for Market Regulation, Chengdu 611731, China; 5 College of Science and Engineering, Flinders University, Adelaide 5042, Australia

**Keywords:** Baijiu fermentation, L-lactic acid-enriched wheat bran *Qu*, L-lactic acid, ester formation, microbial community, light-flavor Baijiu

## Abstract

Lactic acid is a key metabolite in Baijiu fermentation and plays an important role in ester formation and overall flavor development. However, D-lactic acid commonly accumulates in Baijiu and often exceeds L-lactic acid, resulting in low L/D ratios that may negatively affect fermentation performance and sensory quality. Therefore, regulating the optical composition of lactic acid is of significant importance. In this study, an L-lactic acid-enriched wheat bran *Qu* was developed by inoculating *Bacillus cereus* B12 and applied in light-flavor Baijiu fermentation. The preparation conditions were optimized using single-factor experiments and orthogonal experimental design. The optimal conditions were an inoculation level of 3%, moisture content of 55%, cultivation temperature of 37 °C, and cultivation time of 60 h, under which the microbial biomass reached 2.02 × 10^10^ CFU/g. The L-lactic acid-enriched wheat bran *Qu* influenced fermentation characteristics, including substrate utilization and physicochemical parameters. Notably, it significantly increased lactic acid production, particularly L-lactic acid, thereby improving the optical composition of lactic acid. Meanwhile, the concentrations of key ester compounds, including ethyl lactate and ethyl acetate, were significantly enhanced (*p* < 0.05). Microbial community analysis revealed shifts in bacterial composition with enrichment of lactic acid-related genera. These findings demonstrate that L-lactic acid-enriched wheat bran *Qu* can regulate lactic acid production and promote ester formation, providing a potential strategy for improving flavor quality in light-flavor Baijiu fermentation.

## 1. Introduction

Baijiu is one of the most widely consumed distilled spirits in China and is produced through a complex solid-state fermentation process involving diverse microbial communities [1,2]. During fermentation, microorganisms convert carbohydrates and proteins from raw materials into a wide range of metabolites, including organic acids, alcohols, and esters, which collectively contribute to the characteristic aroma and flavor of Baijiu [2,3]. Among the various Baijiu styles, light-flavor Baijiu is characterized by a relatively clean and delicate aroma profile, in which ester compounds, particularly ethyl acetate and ethyl lactate, play key roles in determining overall flavor quality [4,5].

Lactic acid is an important intermediate metabolite in Baijiu fermentation and serves as a key precursor for ester formation [6]. Notably, lactic acid exists in two optical isomers, L-lactic acid and D-lactic acid, which exhibit different sensory properties [6,7]. Previous studies have indicated that L-lactic acid generally contributes a smoother and milder acidity, whereas D-lactic acid is associated with a sharper and more persistent acidic perception [6,7,8]. In addition, the accumulation of D-lactic acid is commonly observed in Baijiu, with its concentration often exceeding that of L-lactic acid in most commercial products, leading to relatively low L/D ratios [6,9]. Excessive D-lactic acid may increase mash acidity, disrupt normal saccharification and fermentation processes, and negatively affect the sensory quality of the final product by imparting a sharp and less desirable taste [6,7,8]. In addition, although D-lactic acid is commonly present in Baijiu and there is currently no national standard specifying the content or L/D ratio of lactic acid enantiomers, excessive accumulation of D-lactic acid has also been associated with potential physiological concerns under certain conditions [7]. Therefore, regulating the optical composition of lactic acid during fermentation may influence the balance of flavor compounds and, consequently, the sensory quality of Baijiu.

Traditional Baijiu fermentation relies on complex microbial ecosystems derived from Daqu and the surrounding environment [3,10]. While this open fermentation system contributes to the diversity of flavor compounds, it also introduces variability in fermentation performance and product quality [11]. In recent years, the use of functional microbial starters has emerged as a promising strategy to regulate microbial succession and improve fermentation consistency [12]. Wheat bran *Qu*, due to its rich nutrient composition, favorable aeration properties, and suitability for microbial growth, has been widely used as a carrier for microorganisms in solid-state fermentation and represents a potential platform for developing functional starter cultures [13,14]. Functional microorganisms with specific metabolic properties may provide a targeted strategy for regulating lactic acid composition and ester formation during Baijiu fermentation. Therefore, identification and application of native functional strains capable of producing high levels of L-lactic acid are important for improving fermentation stability and flavor-related metabolic regulation.

In this study, an L-lactic acid-producing strain (*Bacillus cereus* B12 (*B. cereus* B12)) isolated from pit mud was used to develop a L-lactic acid-enriched wheat bran *Qu* (hereafter referred to as enriched wheat bran *Qu*) [15]. The preparation conditions were optimized through single-factor experiments followed by orthogonal experimental design. The enriched wheat bran *Qu* was then applied to light-flavor Baijiu fermentation to evaluate its effects on fermentation characteristics, lactic acid production, ester formation, and bacterial community structure. This study aims to provide a potential approach for regulating L-lactic acid production and improving flavor formation in light-flavor Baijiu fermentation.

## 2. Materials and Methods

### 2.1. Microorganism and Culture Medium

The L-lactic acid-producing strain used in this study was *B. cereus* B12, which was isolated from pit mud used in Baijiu fermentation and preserved in the laboratory. This strain had been previously identified as a high-yield L-lactic acid producer [15].

Luria–Bertani (LB) medium was used for strain activation and cultivation. The composition of LB medium was as follows (per liter): tryptone 10 g, yeast extract 5 g, sodium chloride 10 g, and distilled water to 1000 mL. For solid medium, agar 20 g/L was added.

### 2.2. Preparation of Seed Culture

LB liquid medium was prepared and sterilized at 121 °C for 20 min and inoculated with *B. cereus* B12 under aseptic conditions. The culture was incubated in a shaking incubator at 37 °C and 150 rpm for 14 h to obtain an actively growing seed culture. The OD_600_ was measured using a UV–visible spectrophotometer, and the culture was adjusted to an OD_600_ of 1.0 prior to use in subsequent experiments [16].

### 2.3. Biomass Quantification of Seed Culture

The viable cell concentration of the seed culture (OD_600_ = 1.0) was determined using the plate count method, as previously described [17]. Serial dilutions of the bacterial suspension were prepared in sterile saline, and aliquots were spread onto LB agar plates. After incubation at 37 °C for 24 h, colonies were counted. Plates containing 30–300 colonies were considered valid, and the results were expressed as colony-forming units (CFU/mL).

### 2.4. Preparation of Enriched Wheat Bran Qu

A solid-state cultivation system was established for the preparation of enriched wheat bran *Qu* using *B. cereus* B12. Wheat bran (170 g) was placed in sterile shallow trays, and sterile distilled water was added to adjust the moisture content to 55% (*w*/*w*). The substrates were covered with sterile gauze and sterilized at 121 °C for 20 min.

After cooling to room temperature, the sterilized wheat bran was inoculated with the seed culture (OD600 = 1.0) at an inoculation level of 10% (*v*/*w*) and thoroughly mixed under aseptic conditions. Control samples were prepared by adding sterile distilled water instead of the inoculum. The substrates were then incubated statically at 37 °C for 3 days for solid-state cultivation. Following cultivation, the samples were dried at 40 °C until the moisture content decreased to ≤12%. The resulting enriched wheat bran *Qu* was vacuum-sealed and stored at 4 °C for subsequent use.

**Figure 1:**
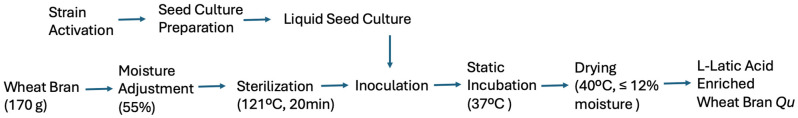
Preparation process of L-lactic acid-enriched wheat bran *Qu.*

The preparation process of enriched wheat bran *Qu* is illustrated in Figure 1. These conditions were used as the baseline for *Qu* preparation and were further optimized as described in Section 2.7.

### 2.5. Biomass Quantification of Enriched Wheat Bran Qu

Microbial biomass was determined using the plate count method and expressed as colony-forming units per gram of wheat bran *Qu* (CFU/g *Qu*). Briefly, 10 g of *Qu* sample was suspended in 90 mL sterile saline and shaken in a water bath shaker at 80 °C and 150 rpm for 30 min to detach microorganisms from the solid substrate [18]. This treatment inactivates heat-sensitive vegetative cells while allowing bacterial endospores to survive and subsequently germinate on agar plates.

The resulting suspension was serially diluted with sterile saline, and aliquots were spread onto LB agar plates. After incubation at 37 °C for 24 h, plates containing 30–300 CFU were selected for enumeration. All measurements were performed in triplicate.

### 2.6. Verification of Dominant Bacterium in Enriched Wheat Bran Qu

To verify whether the dominant bacterium in the enriched wheat bran *Qu* corresponded to the inoculated strain, bacterial 16S rRNA gene sequencing was performed. It should be noted that 16S rRNA gene sequencing provides limited resolution for differentiating closely related members of the *B. cereus* group. Therefore, the present analysis was used to confirm the dominant culturable Bacillus isolate at the genus/species-complex level rather than definitive strain-level identification.

Representative colonies were isolated from LB agar plates and purified by repeated streaking. Genomic DNA was extracted using a Rapid Bacterial DNA Isolation Kit (B518225-0100, Sangon Biotech, Shanghai, China) according to the manufacturer’s instructions.

The extracted DNA was used as a template for PCR amplification of the bacterial 16S rRNA gene using the universal primers 27F (5′-AGAGTTTGATCATGGCTCAG-3′) and 1492R (5′-ACGGTTACCTTGTTACGACTT-3′).

PCR products were first checked by agarose gel electrophoresis followed by sequencing (by Shanghai Sangon Biotech Co., Ltd., Shanghai, China). The obtained sequences were assembled and manually corrected using DNASTAR software (Lasergene 17.3) and compared with reference sequences in the NCBI database using the BLASTn (version 2.14.0+) to determine sequence homology and verify the identity of the dominant bacterial population [19].

### 2.7. Optimization of Enriched Wheat Bran Qu Preparation

The preparation conditions of enriched wheat bran *Qu* was optimized to maximize microbial biomass, which was used as the evaluation index.

#### 2.7.1. Single-Factor Experiments

Single-factor experiments were conducted to evaluate the effects of key cultivation factors including inoculation level (1%, 2%, 3%, 4%, and 5%), cultivation time (24, 36, 48, 60, and 72 h), moisture content (40%, 45%, 50%, 55%, and 60%), and cultivation temperature (31, 33, 35, 37 and 39 °C). During each experiment, one factor was varied while the other parameters were kept constant to evaluate the microbial biomass changes in wheat bran Qu. Enriched wheat bran Qu was prepared following the procedure described in Section 2.4, and microbial biomass was determined as described in Section 2.5.

#### 2.7.2. Orthogonal Experimental Design

Based on the results of the single-factor experiments, an L_9_(3^4^) orthogonal design was applied to further optimize the preparation conditions of enriched wheat bran *Qu*. Inoculation level (A), cultivation time (B), moisture content (C), and cultivation temperature (D) were selected as independent variables, while microbial biomass (CFU/g *Qu*) was used as the response variable.

The factors and levels used in the orthogonal design are presented in Table 1. Enriched wheat bran *Qu* samples were prepared according to the combinations specified in the orthogonal array, and microbial biomass was determined as described above.

The analysis was performed using K and R values to evaluate the relative influence of the investigated factors and to identify an appropriate parameter combination for enriched wheat bran *Qu* preparation.

#### 2.7.3. Validation Experiment

To validate the reliability of the optimized parameter combination, experiments were conducted under the selected cultivation conditions. Enriched wheat bran *Qu* was prepared using the optimized parameters, and microbial biomass was quantified to evaluate the reproducibility and stability of the process. The results were compared with those obtained under the baseline conditions described in Section 2.4.

### 2.8. Application of Enriched Wheat Bran Qu in Light-Flavor Baijiu Fermentation

A laboratory-scale solid-state fermentation model was established based on the traditional light-flavor Daqu Baijiu process. Japonica sorghum was used as the raw material and crushed into 4–8 fragments. The crushed sorghum was hydrated with boiling water at 55% (*w*/*w*, relative to grain weight) and soaked for 20 h, followed by steaming for 1 h until complete gelatinization.

After steaming, additional water was added at 5% (*w*/*w*) of the grain weight according to standard practice [20], and the grains were cooled to room temperature. Traditional light-flavor Daqu was added at 10% (*w*/*w*) of the grain weight. L-lactic acid-enriched wheat bran Qu prepared in Section 2.4 was simultaneously introduced as an auxiliary starter.

**Figure 2:**
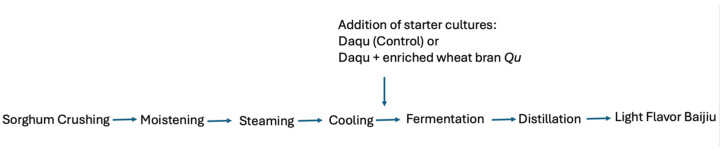
Process Flow Diagram of Ligh-flavor Baijiu Fermentation.

Fermentation was carried out in sealed stainless-steel vessels at approximately 16 °C for 21 days to simulate pit fermentation conditions. After 21 days of fermentation, the fermented grains were distilled to obtain Baijiu according to the traditional light-flavor Baijiu process. The overall fermentation process is illustrated in Figure 2. Due to the practical constraints and relatively high operational cost associated with laboratory-scale solid-state Baijiu fermentation, the fermentation experiment was conducted using a single fermentation vessel per treatment group. The triplicate measurements reported in this study therefore represented analytical/technical replicates rather than independent biological fermentation replicates.

### 2.9. Analysis of Key Physicochemical Properties of Fermented Grains

Fermented grain samples were collected at 5-day intervals during fermentation. Approximately 100 g of material was randomly sampled from three different positions within each fermentation vessel and homogenized prior to analysis. Moisture content was determined by oven drying at 105 °C to constant weight according to GB 5009.3-2016 [21]. Total acidity was determined by titration with 0.1 mol/L NaOH using phenolphthalein as the endpoint indicator [22]. Reducing sugar content was determined using the Fehling titration method (GB/T 5009.7-2016) [23]. Starch content was determined following acid hydrolysis and subsequent Fehling titration [24]. All analyses were conducted in triplicate.

### 2.10. Analysis of Lactic Acid and Optical Purity

L- and D-lactic acid were analyzed using high-performance liquid chromatography (HPLC) as previously described [9]. Analyses were performed using an HPLC system (K2025, Shandong Wukong Instrument Co., Ltd., Dezhou, China) equipped with a UV detector.

Chiral separation was achieved using an FMB-AAOA3-EONU Chiral AAOA column (5 × 4.6 mm, 3 μm; Guangzhou Philomen Scientific Instrument Co., Ltd., Guangzhou, China). The mobile phase consisted of 0.2 mmol/L CuSO_4_–isopropanol (95:5, *v*/*v*). Detection was carried out at 254 nm with a column temperature of 25 °C and an injection volume of 10 μL. Quantification was performed using external calibration with L- and D-lactic acid standards.

Total lactic acid was determined using HPLC (K2025, Shandong Wukong Instrument Co., Ltd., China) equipped with a C18 column (5 μm, 4.6 × 250 mm; Shimadzu Enterprise Management Co., Ltd., Shanghai, China). The mobile phase consisted of KH_2_PO_4_–methanol (95:5, *v*/*v*), and detection was performed at 215 nm. Optical purity was calculated as the proportion of L-lactic acid relative to total lactic acid. All measurements were conducted in triplicate.

### 2.11. Analysis of Ester Compounds

After 21 days of fermentation, fermented grains were distilled to obtain Baijiu. The distillate fraction collected between 50 and 200 mL was retained as the analytical sample and stored at 4 °C prior to analysis.

Ethyl lactate and ethyl acetate were quantified by gas chromatography as previously described [25], using a GC system (GC 8890, Agilent Technologies, Santa Clara, CA, USA) equipped with a flame ionization detector (FID) and a capillary column (50 m × 0.32 mm × 1.0 μm; Agilent Technologies, Santa Clara, CA, USA).

Prior to analysis, Baijiu samples were adjusted to 50% (*v*/*v*) with ultrapure water and filtered through a 0.22 μm membrane. Mixed standard solutions of ethyl lactate and ethyl acetate were prepared in 50% ethanol for external calibration.

Nitrogen was used as the carrier gas at a flow rate of 1.0 mL/min with a split ratio of 10:1. The injector and detector temperatures were both set at 220 °C. The oven temperature program was as follows: 60 °C (3 min), increased to 90 °C at 3 °C/min (held for 5 min), then to 140 °C at 3 °C/min (held for 2 min), followed by 150 °C (held for 5 min), and finally increased to 220 °C at 5 °C/min (held for 10 min).

Quantification was performed using external calibration with ethyl lactate and ethyl acetate standards. All measurements were conducted in triplicate.

### 2.12. Bacterial Community Analysis of Fermented Grains

Microbial community analysis of fermented grains was performed using high-throughput sequencing of bacterial 16S rRNA genes.

Fermented grain samples were suspended in sterile distilled water and shaken to detach microorganisms from the solid matrix. The resulting suspension was centrifuged to collect microbial biomass for DNA extraction.

Total microbial DNA was extracted using a commercial DNA extraction kit according to the manufacturer’s instructions. DNA concentration and integrity were evaluated prior to PCR amplification.

The bacterial 16S rRNA gene was amplified using universal primers. The resulting amplicons were sequenced using a high-throughput sequencing platform by Majorbio Bio-Pharm Technology Co., Ltd. (Shanghai, China).

Raw sequencing reads were processed using standard bioinformatic pipelines. Briefly, raw paired-end reads were demultiplexed and quality-filtered (bases with quality score < Q20 were trimmed; reads shorter than 200 bp were discarded). Paired-end reads were merged using FLASH. Chimeric sequences were identified and removed using UCHIME. Sequences were clustered into operational taxonomic units (OTUs) at 97% sequence identity using UPARSE, with one representative sequence selected per OUT. Taxonomic assignment of the resulting sequences was performed against the SILVA 138.2 bacterial 16S rRNA gene reference database to determine the microbial community composition.

### 2.13. Statistical Analysis

All measurements were performed in triplicate unless otherwise stated, and the results are presented as mean ± standard deviation (SD). Statistical analyses were performed using SPSS 19.0 software (SPSS Inc., Chicago, IL, USA). Differences among treatments were evaluated using one-way analysis of variance (ANOVA) followed by Duncan’s multiple range test. A *p*-value < 0.05 was considered statistically significant.

Graphs were generated using Origin and Microsoft Excel. Orthogonal experimental results were analyzed using Orthogonal Design Assistant II (Version 3.1).

## 3. Results

### 3.1. Development and Optimization of Enriched Wheat Bran Qu

To develop the enriched wheat bran *Qu*, *B. cereus* B12 was inoculated into a wheat bran-based solid-state system. Process optimization was then performed to maximize microbial biomass by systematically evaluating key cultivation parameters, including inoculation level, temperature, time, and moisture content, using single-factor experiments followed by a multivariate optimization design, and the optimal preparation conditions were subsequently determined and validated. The investigated ranges for each factor were selected based on preliminary trials and previously reported conditions for solid-state fermentation using wheat bran or related cereal substrates. Inoculation levels of 1–5% were examined to identify an appropriate inoculum level while avoiding excessive inoculation that may not further promote biomass accumulation [26,27]. The cultivation temperature range of 31–39 °C was centred around 35–37 °C, which is commonly used for mesophilic *Bacillus* cultivation in solid-state fermentation [28,29]. The cultivation time range of 24–72 h was selected to capture biomass accumulation during the main growth phase [30,31]. Moisture content was varied from 40% to 60% to cover the commonly reported range suitable for microbial growth and enzyme production in wheat bran-based solid-state systems [15,32,33].

#### 3.1.1. Influence of Inoculation Level on Microbial Biomass During Qu Preparation

**Figure 3:**
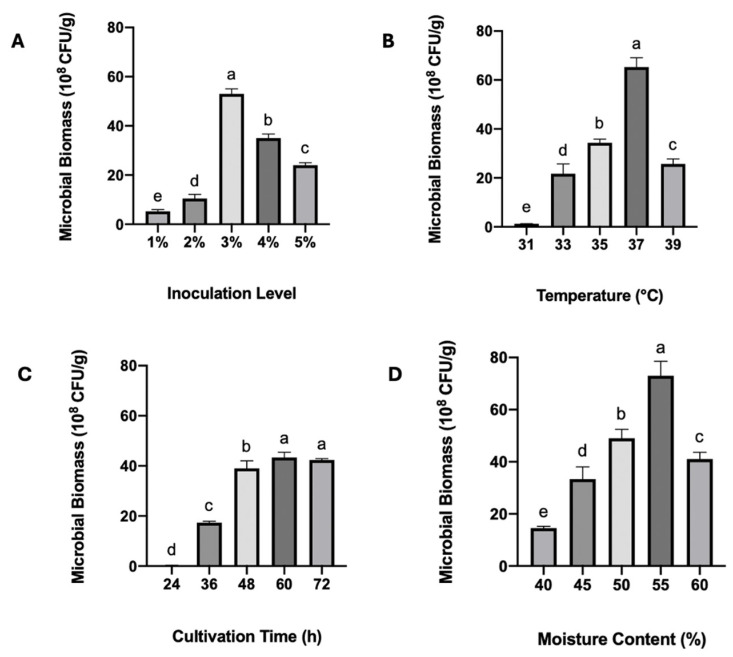
Effect of single-factor cultivation conditions on the microbial biomass in enriched wheat bran *Qu* preparation: (**A**) different inoculation levels at fixed temperature (37 °C), cultivation time (72 h) and moisture content (55%), (**B**) different cultivation temperature at fixed inoculation level (3%), cultivation time (72 h) and moisture content (55%), (**C**) different cultivation time at fixed inoculation level (3%), temperature (37 °C) and moisture content (55%), (**D**) different moisture content at fixed inoculation level (3%), temperature (37 °C) and cultivation time (72 h). Different letters above the bars indicate significant differences among groups (*p* < 0.05).

The inoculation level is an important factor influencing microbial growth during the preparation of enriched wheat bran *Qu*. As shown in Figure 3A, the microbial biomass increased initially and then decreased with increasing inoculation level.

When the inoculation level was 3%, the microbial biomass reached 5.3 × 10^9^ CFU/g, which was significantly higher than those observed in the other groups (*p* < 0.05). In contrast, the biomass obtained at 1% inoculation was only 5.2 × 10^8^ CFU/g, indicating insufficient microbial growth under low inoculation conditions.

However, when the inoculation level increased to 5%**,** the biomass decreased markedly, possibly due to intensified competition for nutrients and space among microbial cells during solid-state cultivation. Therefore, an inoculation level of approximately 3% was considered appropriate for the preparation of enriched wheat bran *Qu*.

#### 3.1.2. Influence of Cultivation Temperature on Microbial Biomass During Qu Preparation

Temperature is a critical factor influencing microbial metabolism during solid-state fermentation. As shown in Figure 3B, the microbial biomass increased with increasing temperature from 31 °C to 37 °C, reaching a maximum value of 6.57 × 10^9^ CFU/g at 37 °C, which was significantly higher than those observed at other temperatures (*p* < 0.05).

When the cultivation temperature further increased to 39 °C, the biomass decreased markedly to approximately 2.75 × 10^9^ CFU/g. This reduction may be associated with the inhibitory effect of elevated temperature on microbial growth under solid-state cultivation conditions. Therefore, 37 °C was considered the optimal cultivation temperature for the preparation of enriched wheat bran *Qu*.

Overall, the observed differences in microbial biomass should be interpreted primarily as general trends in microbial growth and enrichment efficiency during wheat bran Qu preparation, rather than as emphasizing minor numerical differences between individual treatments.

#### 3.1.3. Influence of Cultivation Time on Microbial Biomass During Qu Preparation

Cultivation time is an important factor affecting microbial growth during the preparation of enriched wheat bran *Qu*. As shown in Figure 3C, the microbial biomass increased with increasing cultivation time during the early stage of solid-state cultivation.

At 48 h, the microbial biomass reached 3.9 × 10^9^ CFU/g, and continued to increase slightly, reaching a maximum value of 4.33 × 10^9^ CFU/g at 60 h. However, when the cultivation time was extended to 72 h, no significant further increase in biomass was observed, and the biomass remained at a level comparable to that at 60 h.

The absence of biomass further increases at prolonged cultivation time may be related to nutrient depletion or reduced water availability in the solid substrate. Therefore, 60 h was considered the appropriate cultivation time for the preparation of enriched wheat bran *Qu*.

#### 3.1.4. Influence of Moisture Content on Microbial Biomass During Qu Preparation

Moisture content is a key factor affecting microbial growth during solid-state fermentation. As shown in Figure 3D, the microbial biomass increased with increasing moisture content from 40% to 55% and then decreased when the moisture content was further increased.

The maximum biomass was obtained at 55% moisture content, reaching 7.30 × 10^9^ CFU/g, which was significantly higher than that observed under other moisture conditions (*p* < 0.05). In contrast, when the moisture content was 40%, the biomass was only 1.46 × 10^9^ CFU/g, indicating insufficient water availability for microbial growth.

When the moisture content increased to 60%, the biomass decreased compared with that at 55%, suggesting that excessive moisture may negatively affect oxygen transfer and microbial activity during solid-state cultivation. Therefore, 55% moisture content was considered optimal for the preparation of enriched wheat bran *Qu*.

#### 3.1.5. Optimization of Qu Preparation Conditions Using Orthogonal Experimental Design

To further optimize the preparation conditions of enriched wheat bran *Qu*, an L_9_(3^4^) orthogonal experimental design was employed based on the results of the single-factor experiments. Inoculation level (A), cultivation time (B), moisture content (C), and cultivation temperature (D) were selected as independent variables, while the microbial biomass in enriched wheat bran *Qu* was used as the evaluation index.

The factors and their levels are presented in Table 1, and the experimental results are summarized in Table 2. One experimental run (Run 3) showed a notably low biomass value, which may be attributed to the unfavorable combination of high temperature (39 °C), high moisture content (60%), prolonged cultivation time (72 h), and low inoculation level (1%), conditions that are generally unfavorable for microbial growth during solid-state fermentation.

Range analysis of the orthogonal experimental design is presented in Table S1. K values represent the sum of experimental results at each factor level, and the range (R) value was used to evaluate the relative influence of each factor. The results indicated that the influence of the four factors on microbial biomass followed the order: D > C > A > B, indicating that cultivation temperature had the greatest effect on biomass, followed by moisture content, inoculation level, and cultivation time.

According to the analysis, the optimal parameter combination was A_2_B_2_C_2_D_2_**,** corresponding to inoculation level 3%, cultivation time 60 h, moisture content 55% and cultivation temperature 37 °C. These conditions were therefore selected as the optimal parameters for the preparation of enriched wheat bran *Qu*.

#### 3.1.6. Validation of Optimized Preparation Conditions

To verify the reliability of the optimized parameters obtained from the previous section, enriched wheat bran *Qu* was prepared under the optimal conditions (A_2_B_2_C_2_D_2_), and the resulting biomass was compared with that obtained under the baseline preparation conditions.

**Figure 4:**
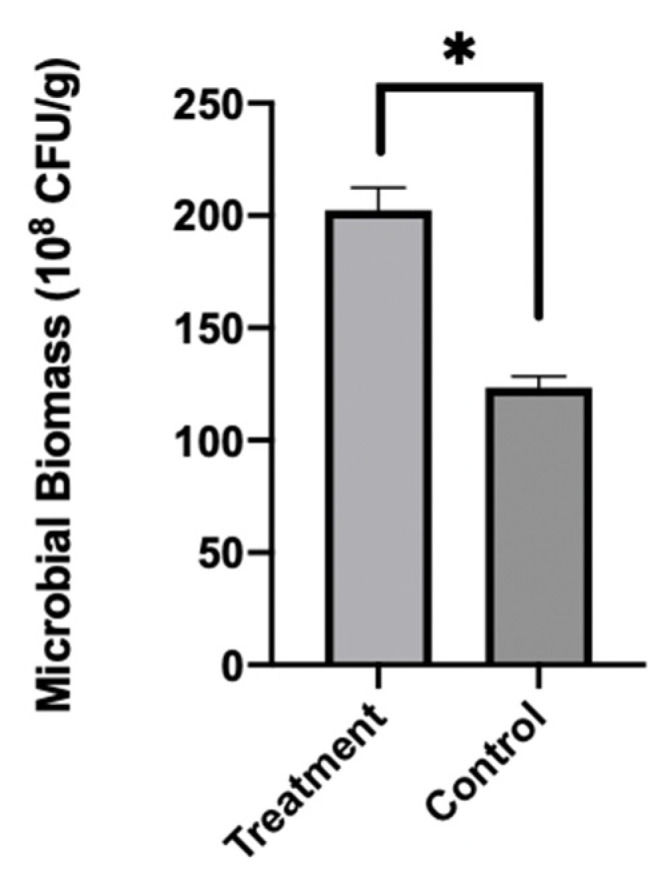
Validation of optimized preparation conditions for enriched wheat bran *Qu*. *, *p* < 0.05.

As shown in Figure 4, the microbial biomass in the optimized group reached 2.02 × 10^10^ CFU/g, which was significantly higher than that in the control group (1.23 × 10^10^ CFU/g, *p* < 0.05). These results confirm that the optimized preparation conditions effectively improved the microbial biomass in enriched wheat bran *Qu*.

### 3.2. Identification of the Dominant Bacterium in Enriched Wheat Bran Qu

To verify whether the inoculated strain (*B. cereus* B12) dominated the microbial population in enriched wheat bran *Qu*, representative colonies isolated from the *Qu* were subjected to 16S rRNA gene sequencing. The obtained sequences were compared with reference sequences in the NCBI database using the BLAST program (BLASTN 2.14.0+).

**Figure 5:**
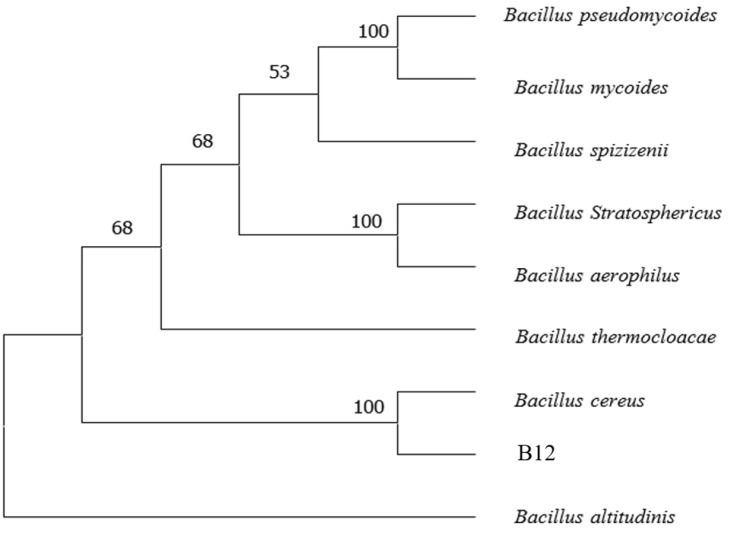
Phylogenetic tree of the dominant bacterium.

A phylogenetic tree was constructed using MEGA 7, and the result is shown in Figure 5. The isolated strain clustered with *B. cereus* and exhibited 100% sequence similarity with the reference strain.

These results indicate that the dominant bacterial population in the prepared enriched wheat bran *Qu* was consistent with the inoculated Bacillus isolate at the *B. cereus* group/species-complex level.

### 3.3. Effects of Enriched Wheat Bran Qu on Fermentation Characteristics

To evaluate the effects of enriched wheat bran *Qu* on the fermentation characteristics of light-flavor Baijiu, key physicochemical parameters of fermented grains were systematically monitored throughout fermentation, including starch and reducing sugar contents, acidity, moisture, and temperature.

#### 3.3.1. Changes in Substrate Utilization (Starch and Reducing Sugars)

**Figure 6:**
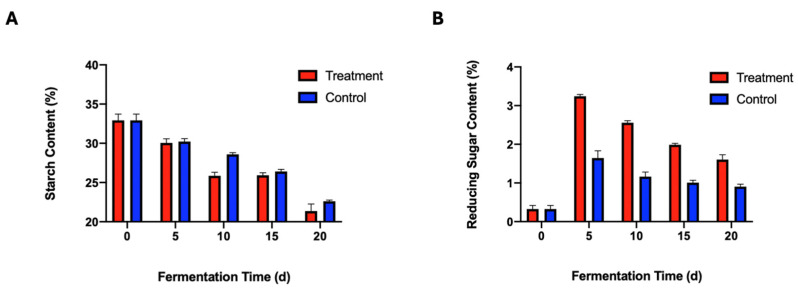
Changes in substrate utilization during light-flavor Baijiu fermentation: (**A**) starch content, and (**B**) reducing sugar content.

The changes in starch and reducing sugar contents during light-flavor Baijiu fermentation are shown in Figure 6A and Figure 6B, respectively.

As shown in Figure 6A, the starch content of fermented grains decreased continuously during fermentation in both the control and experimental groups. In the early stage of fermentation (0–10 d), the starch content in the control group decreased more rapidly than that in the experimental group. During the middle stage (10–15 d), the starch content in the experimental group remained relatively stable, whereas that in the control group continued to decline. After 15 d, the starch contents of both groups decreased rapidly and exhibited similar decreasing trends. These results suggest that enriched wheat bran *Qu* had limited influence on starch degradation during light-flavor Baijiu fermentation.

The changes in reducing sugar content are presented in Figure 6B. At the beginning of fermentation (0 d), the reducing sugar contents of the two groups were low and nearly identical. As fermentation progressed, the reducing sugar content in the experimental group increased rapidly and reached a maximum value of 3.0–3.5% at 5 d, followed by a gradual decrease. In contrast, the control group showed only a slight increase, reaching approximately 1.5% at 5 d, and then remained relatively stable or slightly decreased. These results indicate that enriched wheat bran *Qu* enhanced sugar metabolism during fermentation.

#### 3.3.2. Changes in Fermentation Environment (Acidity, Moisture, and Temperature)

**Figure 7:**
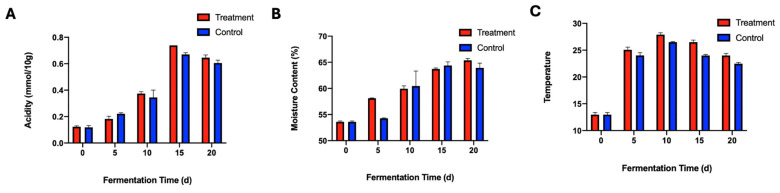
Changes in fermentation environment during light-flavor Baijiu fermentation: (**A**) acidity, (**B**) moisture, and (**C**) temperature.

The changes in acidity, moisture content, and fermentation temperature during light-flavor Baijiu fermentation are shown in Figure 7.

As shown in Figure 7A, the acidity of fermented grains in both the control and experimental groups gradually increased during fermentation. During the early fermentation stage (0–10 d), the experimental group showed a slightly faster increase compared with the control group. During the middle stage of fermentation (10–15 d), the acidity in both groups continued to increase and reached relatively high levels. In the later stage of fermentation, the acidity of both groups decreased slightly and then increased again toward the end of fermentation. Overall, the acidification trends of the two groups were similar, indicating that enriched wheat bran *Qu* did not significantly alter the acidification process. The gradual increase in acidity during the early and middle stages of fermentation was likely associated with active microbial metabolism and organic acid production. The slight decrease in acidity observed during the later fermentation stage may be related to reduced acid production following substrate depletion, as well as the conversion of organic acids into ester compounds through esterification reactions during fermentation.

The moisture content of fermented grains showed moderate fluctuations during fermentation (Figure 7B). During the early stage (0–10 d), the moisture contents of both groups increased, with the experimental group showing a slightly larger increase. In the middle stage (10–15 d), the moisture content in the experimental group exceeded that of the control group. During the later stage (15–20 d), the moisture content of the experimental group decreased slightly, whereas that of the control group remained relatively stable. Toward the end of fermentation (20–25 d), the moisture contents of the two groups became similar and remained relatively stable. The observed fluctuations in moisture content may be associated with microbial metabolic activity, substrate degradation, and water redistribution during solid-state fermentation. Appropriate moisture levels are important for maintaining microbial growth and metabolic activity throughout the fermentation process.

The changes in fermentation temperature are shown in Figure 7C. At the beginning of fermentation (0 d), the temperatures of both groups were relatively low and similar. During the early stage of fermentation, the temperature increased gradually and reached a peak at around 10 d, with the experimental group reaching approximately 28 °C and the control group approximately 26 °C. After the temperature peak, the temperatures in both groups gradually decreased. By the end of fermentation (25 d), the temperatures declined to approximately 23 °C in the experimental group and 22 °C in the control group. The increase in temperature during the early fermentation stage was likely associated with active microbial growth and metabolic heat generation. The subsequent decline in temperature during the later stage may reflect reduced microbial activity and substrate utilization as fermentation progressed.

Overall, these results indicate that enriched wheat bran *Qu* did not substantially affect the physicochemical environment of light-flavor Baijiu fermentation, although minor variations in fermentation dynamics were observed. These results indicate that enriched wheat bran Qu did not substantially alter the physicochemical environment of light-flavor Baijiu fermentation, although minor variations in fermentation dynamics associated with microbial metabolic activity were observed.

### 3.4. Effects of Enriched Wheat Bran Qu on Lactic Acid Profiles and Ester Formation

To further evaluate the effects of enriched wheat bran *Qu* on lactic acid production and ester formation during light-flavor Baijiu fermentation, the variations in total lactic acid, L-lactic acid, and key ester compounds were systematically analyzed.

#### 3.4.1. Changes in Total Lactic Acid During Fermentation

**Figure 8:**
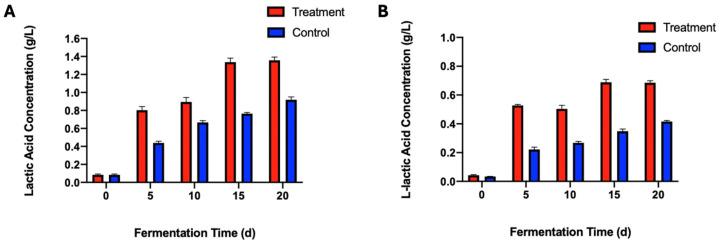
Changes in lactic acid profiles during light-flavor Baijiu fermentation: (**A**) lactic acid concentration, and (**B**) L-lactic acid concentration.

The changes in lactic acid concentration during light-flavor Baijiu fermentation are shown in Figure 8A.

As fermentation progressed, the lactic acid concentration in both the control and experimental groups increased gradually. During the early fermentation stage (0–5 d), the experimental group exhibited a faster increase in lactic acid concentration and showed significantly higher levels than the control group. From 5 to 15 d, the lactic acid concentrations in both groups continued to increase, although the rate of increase in the experimental group decreased.

After approximately 15 d, the lactic acid concentration in the experimental group reached a relatively stable level of about 1.4 g/L, whereas the control group continued to increase slowly and reached approximately 0.8 g/L at 20 d.

These results indicate that the addition of enriched wheat bran *Qu* enhanced lactic acid accumulation during light-flavor Baijiu fermentation.

#### 3.4.2. Changes in L-Lactic Acid During Fermentation

The changes in L-lactic acid concentration during light-flavor Baijiu fermentation are shown in Figure 8B.

As fermentation progressed, the L-lactic acid concentration in both the control and experimental groups increased gradually. During the early fermentation stage (0–5 d), the experimental group exhibited a markedly faster increase in L-lactic acid concentration compared with the control group, indicating enhanced L-lactic acid production at this stage.

From 5 to 10 d, the L-lactic acid concentration in the experimental group remained at relatively high levels with minor fluctuations, whereas the control group showed a slower but continuous increase. During the middle fermentation stage (10–15 d), the L-lactic acid concentration in the experimental group continued to increase and gradually approached a stable level, while the control group increased at a lower rate.

In the later stage of fermentation (15–20 d), the L-lactic acid concentration in the experimental group remained relatively stable, whereas the control group continued to increase slowly. Throughout the fermentation process, the L-lactic acid concentration in the experimental group remained consistently higher than that in the control group.

The corresponding changes in D-lactic acid concentration during fermentation are presented in the Supplementary Materials (Figure S1). Although the enriched wheat bran Qu markedly promoted L-lactic acid accumulation, D-lactic acid remained detectable throughout the fermentation process and still accounted for approximately 25–50% of the total lactic acid concentration at different fermentation stages. These results suggest that the enriched wheat bran Qu shifted the optical composition of lactic acid toward L-lactic acid dominance; however, complete suppression of D-lactic acid production may be difficult in the complex microbial ecosystem of traditional Baijiu fermentation.

These results demonstrate that the addition of enriched wheat bran *Qu* enhanced L-lactic acid accumulation during light-flavor Baijiu fermentation.

#### 3.4.3. Optical Purity of Lactic Acid

**Figure 9:**
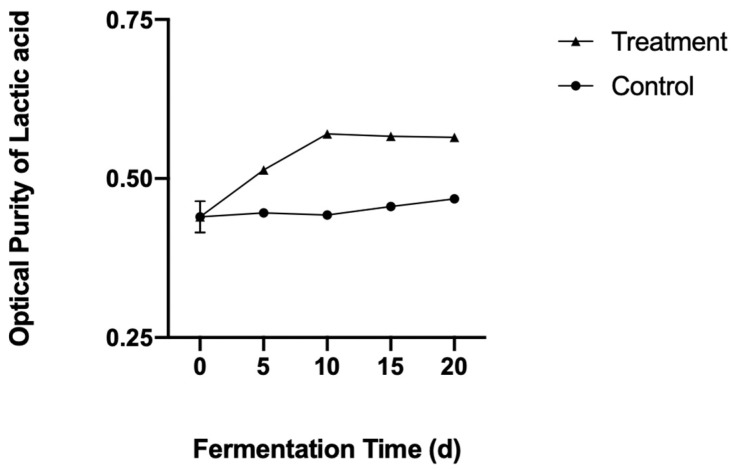
Changes in optical purity during light-flavor Baijiu fermentation.

The optical purity of lactic acid, defined as the proportion of L-lactic acid in total lactic acid, was evaluated to assess the effect of enriched wheat bran Qu on lactic acid composition during fermentation. As shown in Figure 9, the optical purity in both groups increased during the early stage of fermentation and gradually stabilized thereafter.

Notably, the enriched wheat bran Qu group consistently exhibited higher optical purity compared to the control throughout the fermentation process. The difference became more pronounced after 5 days of fermentation and remained stable at later stages. This indicates that the increase in L-lactic acid was more pronounced relative to D-lactic acid in the enriched wheat bran *Qu* group, resulting in an improved L/D ratio.

Although D-lactic acid was also present during fermentation, the relative enhancement of L-lactic acid dominated the overall compositional shift. The improved optical purity suggests that the enriched wheat bran *Qu* effectively regulated lactic acid stereoisomer composition toward a more favorable profile, which may contribute to enhanced flavor development in light-flavor Baijiu.

#### 3.4.4. Changes in Ethyl Lactate and Ethyl Acetate Formation in Light-Flavor Baijiu

**Figure 10:**
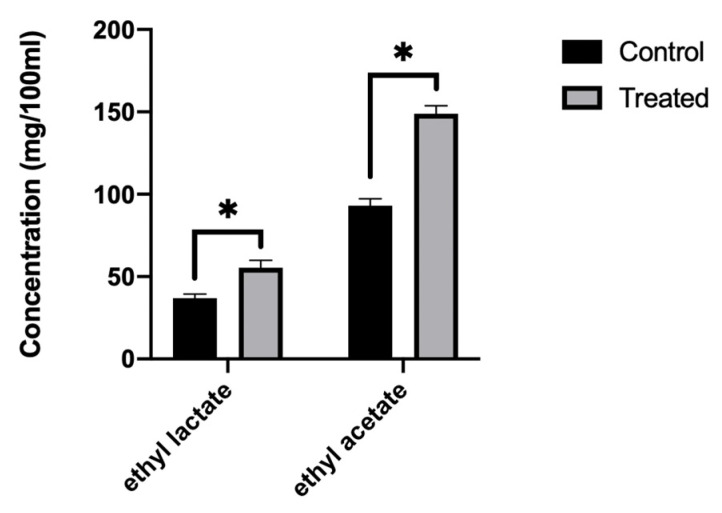
Concentration of ethyl lactate and ethyl acetate in light-flavor Baijiu. * *p* < 0.05.

The concentrations of ethyl lactate and ethyl acetate in light-flavor Baijiu are shown in Figure 10. The addition of enriched wheat bran *Qu* significantly increased the concentrations of both ester compounds compared with the control group (*p* < 0.05). In the experimental group, the ethyl acetate concentration reached 148.96 mg/100 mL, whereas the control group contained 93.01 mg/100 mL. Similarly, the ethyl lactate concentration increased to 55.44 mg/100 mL in the experimental group, compared with 36.93 mg/100 mL in the control group. This increase in ethyl lactate is consistent with the higher lactic acid levels observed during fermentation. These results indicate that the addition of enriched wheat bran *Qu* enhanced the formation of key ester compounds during light-flavor Baijiu production.

### 3.5. Effects of Enriched Wheat Bran Qu on Bacterial Community Structure During Fermentation

#### 3.5.1. Alpha Diversity of Bacterial Communities Under Enriched Wheat Bran Qu and Control Conditions

The bacterial α-diversity indices of fermented grain samples are summarized in Table 3. The sequencing coverage of all samples exceeded 99%, indicating that the sequencing depth was sufficient to capture the majority of bacterial diversity in the fermented grains.

Compared with the control group (F1), the experimental group (F2) exhibited higher ACE, Chao1, and Shannon indices, suggesting that the addition of enriched wheat bran *Qu* increased both the richness and diversity of bacterial communities in the fermented grains.

#### 3.5.2. Genus-Level Bacterial Community Composition Under Enriched Wheat Bran Qu and Control Conditions

**Figure 11:**
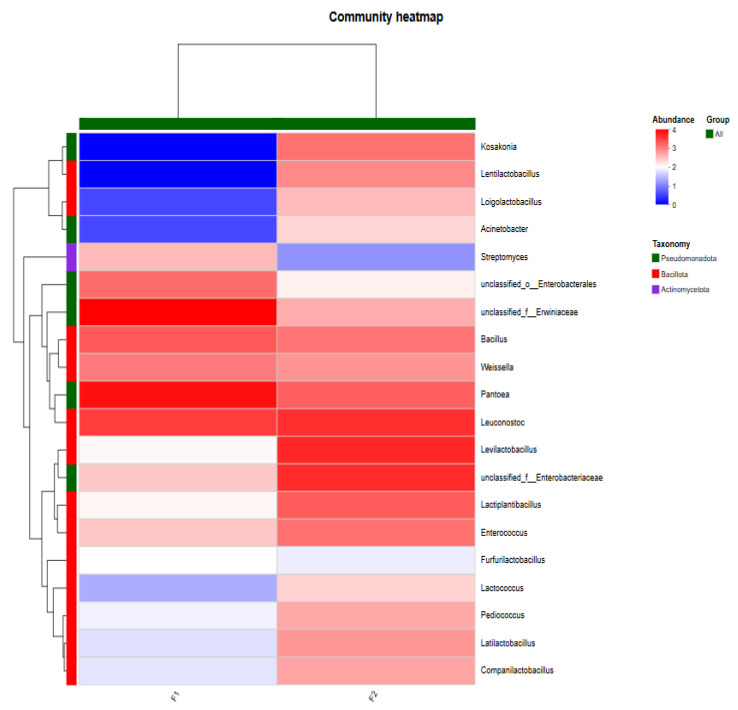
Heatmap of bacterial community composition.

The genus-level bacterial community composition of fermented grains is presented in Figure 11.

In the control group (F1), the dominant bacterial genera included *Pantoea*, *Leuconostoc*, *Bacillus*, *Weissella*, *Enterococcus*, and several members of the *Enterobacteriaceae* family. With the addition of enriched wheat bran *Qu* (F2), noticeable changes in microbial composition were observed. The relative abundances of several lactic acid bacteria, including *Levilactobacillus*, *Lactiplantibacillus*, and *Lentilactobacillus*, increased in the inoculated fermentation system. In addition, genera such as *Kosakonia* were also detected at higher levels in the inoculated samples.

Overall, the addition of enriched wheat bran *Qu* altered the bacterial community structure of the fermented grains. The enrichment of lactic acid-related bacterial genera may contribute to enhanced lactic acid metabolism during fermentation.

## 4. Discussion

### 4.1. Optimization of Enriched Wheat Bran Qu Preparation

The preparation conditions of enriched wheat bran *Qu* had a significant impact on microbial biomass, highlighting the importance of process optimization in solid-state fermentation systems. Among the investigated factors, moisture content plays a critical role by regulating oxygen transfer, substrate accessibility, and microbial metabolic activity [34,35,36]. In this study, the optimal moisture content was determined to be 55%, which is consistent with previous reports indicating that moderate moisture levels can support microbial growth while maintaining adequate aeration during solid-state fermentation.

Temperature was another key factor influencing biomass accumulation. The highest microbial biomass was observed at 37 °C, which is close to the optimal growth temperature reported for many *Bacillus* species. At higher temperatures, microbial growth may be inhibited due to thermal stress and reduced enzymatic activity, leading to decreased biomass formation [37,38]. These results collectively demonstrate that appropriate control of moisture and temperature is essential for achieving efficient biomass production in enriched wheat bran *Qu* preparation.

### 4.2. Impact of Enriched Wheat Bran Qu on Fermentation Characteristics

The addition of enriched wheat bran *Qu* influenced several physicochemical parameters during light-flavor Baijiu fermentation. Changes in acidity, moisture, and fermentation temperature reflected the metabolic activity of the microbial community during fermentation.

Although the addition of wheat bran *Qu* did not significantly alter the overall trends of starch degradation, the reducing sugar content increased more rapidly in the inoculated fermentation system. This phenomenon may be attributed to the enzymatic activities of *Bacillus* species, which are known to produce various hydrolytic enzymes, including amylases and proteases [39,40]. These enzymes can promote the hydrolysis of starch and enhance the availability of fermentable sugars for microbial metabolism [41].

### 4.3. Regulation of Lactic Acid Production During Fermentation

Lactic acid is an important intermediate metabolite in Baijiu fermentation and plays a key role in flavor formation. In this study, the addition of enriched wheat bran *Qu* significantly increased the concentration of lactic acid during fermentation, particularly the L-lactic acid isomer.

Previous studies have shown that L- and D-lactic acid exhibit different sensory characteristics in alcoholic beverages. L-lactic acid generally produces a smoother and milder acidic taste, whereas D-lactic acid tends to contribute a sharper acidity [6,7,8]. Therefore, increasing the proportion of L-lactic acid may help improve the sensory balance of Baijiu [6,7,8].

The results of this study suggest that the addition of enriched wheat bran *Qu* may regulate the metabolic pathways involved in lactic acid production during fermentation. This regulation could be associated with interactions between *Bacillus* species and lactic acid bacteria within the fermentation ecosystem.

### 4.4. Mechanisms of Enhanced Ester Formation Induced by Enriched Wheat Bran Qu

Ester compounds are key contributors to the aroma and flavor of Baijiu, among which ethyl lactate and ethyl acetate are particularly important in light-flavor Baijiu [4,5]. In the present study, both esters were detected at significantly higher concentrations in the fermentation system supplemented with enriched wheat bran *Qu*, indicating a positive role of this starter in modulating ester formation.

The enhanced production of ethyl lactate is likely associated with the elevated lactic acid levels observed during fermentation, as ethyl lactate is primarily formed through the esterification of lactic acid with ethanol [42]. Therefore, the increased availability of lactic acid may have provided a greater substrate pool for esterification, thereby promoting ethyl lactate formation. In addition, the increase in ethyl acetate suggests that enriched wheat bran *Qu* may also influence broader esterification processes, potentially through its impact on microbial activity and metabolic interactions [43,44].

Overall, these findings suggest that the addition of enriched wheat bran *Qu* not only altered organic acid production but also facilitated the formation of key ester compounds, which may contribute to flavor formation in light-flavor Baijiu.

### 4.5. Microbial Basis of Fermentation Changes

High-throughput sequencing revealed that the addition of enriched wheat bran *Qu* altered the bacterial community structure of fermented grains. In particular, several lactic acid-producing genera, including *Levilactobacillus*, *Lactiplantibacillus*, and *Lentilactobacillus*, showed increased relative abundance in the inoculated fermentation system.

These microorganisms are known to participate in carbohydrate metabolism and organic acid production during fermentation [45,46,47]. Their enrichment may explain the increased production of lactic acid and L-lactic acid observed in this study. The selective enrichment of these LAB genera in F2 may be attributed to the enzymatic activity of *B. cereus* B12, which increased reducing sugar availability in the early fermentation stage (Figure 6B) and thereby created a more favorable substrate environment for fastidious LAB, as well as to the early accumulation of lactic acid in F2 (Figure 8), which selectively favored acid-tolerant genera while suppressing acid-sensitive *Enterobacteriaceae* that dominated F1. The decreased relative abundance of *Pantoea* and *Enterobacteriaceae* members in F2 is consistent with this competitive exclusion under the more acidic conditions established early in fermentation. The increase in *Kosaknoia*, a genus associated with carbohydrate utilization and biomass degradation, may reflect its capacity to utilize the complex substrates present in the fermentation system, though its specific functional role warrants further investigation. In addition, the higher alpha diversity observed in F2 suggests that the introduction of enriched wheat bran *Qu* not only enriched specific functional genera but also broadened the overall bacterial community structure, which may contribute to a more complex metabolic network during fermentation.

Overall, the results suggest that enriched wheat bran *Qu* can reshape the microbial ecosystem of fermented grains and thereby regulate metabolic pathways involved in organic acid and ester formation.

Given that B. cereus B12 was used as the functional strain in the present study, its potential biosafety implications should also be considered. Although *B. cereus* is recognized as an opportunistic foodborne pathogen, its pathogenic potential is highly strain-specific and is determined by the presence and functional expression of particular toxin-encoding genes rather than by species membership alone [48]. The principal virulence determinants responsible for food poisoning—the emetic toxin cereulide (*ces*), non-hemolytic enterotoxin (Nhe), hemolysin BL (Hbl), and cytotoxin K (CytK)—are distributed unevenly across strains within the *B. cereus* group, and their absence in a given isolate is associated with negligible pathogenic risk [49]. The *B. cereus* group therefore encompasses strains ranging from highly pathogenic to entirely non-pathogenic, and species-level classification alone cannot be used to predict safety [50]. Strain B12 used in the present study was isolated directly from Zaopei, the fermented grain environment of Baijiu, where *B. cereus* is a naturally dominant and well-documented member of the bacterial community [15,51,52]. In addition, strain B12 has previously been isolated, characterized, and applied in a peer-reviewed Baijiu fermentation study focused on L-lactic acid and ethyl lactate production, confirming its established role as a native functional microorganism in this fermentation system [15]. The strain was selected in the present study based on its functional performance in promoting L-lactic acid production and regulating ester formation during Baijiu fermentation. The application of *B. cereus*-group strains at high concentrations in food and feed contexts has been demonstrated to be safe when the strain is confirmed non-toxigenic. Williams et al. (2009) conducted a comprehensive safety review of *B. cereus* var. *toyoi* (Toyocerin), a non-toxigenic *B. cereus*-group strain used as a commercial feed probiotic, and reported no adverse effects in acute, subchronic, or chronic toxicity studies in rodents at up to 3 × 10^11^ spores/kg body weight/day, and no adverse effects in an eight-day human clinical trial at 1 × 10^10^ spores/kg body weight/day, demonstrating that high-count application of non-toxigenic *B. cereus*-group strains does not inherently constitute a safety risk [53]. Furthermore, consumer exposure to viable *B. cereus* cells via the final distilled product is negligible. Takahashi et al. (2021) demonstrated experimentally that *B. cereus* spores do not proliferate during the manufacturing of fermented alcoholic beverages, that emetic toxin was not detected in any product samples, and concluded that the microbiological safety risk in such products is negligible [54]. Given that Baijiu undergoes a distillation step at high temperature that sake does not, elimination of viable bacterial cells and potential thermolabile toxins from the final product is expected to be complete. Taken together, the native origin of strain B12 from the Baijiu fermentation environment, the established precedent for non-toxigenic *B. cereus*-group strains in food and feed applications, and the safety barrier provided by the distillation process collectively indicate that the application of strain B12 in the present study does not pose a significant biosafety risk to consumers. However, further biosafety evaluation of strain B12, including toxin-related characterization and safety assessment under industrial application conditions, would still be valuable before large-scale industrial application.

### 4.6. Limitations and Future Perspectives

Although this study demonstrated that the incorporation of an L-lactic acid-producing strain can enhance L-lactic acid levels during light-flavor Baijiu fermentation, several limitations should be acknowledged.

Firstly, the experiments were conducted at the laboratory scale, and the performance and stability of the enriched wheat bran *Qu* under industrial fermentation conditions remain to be validated. In addition, due to the practical constraints and relatively high operational cost associated with laboratory-scale solid-state Baijiu fermentation, the fermentation experiment was conducted using a single fermentation vessel per treatment group. Therefore, the reported replicate measurements represent analytical/technical replicates rather than independent biological fermentation replicates. Although this limitation does not affect the observed trends, future studies incorporating independent biological fermentation replicates would further strengthen the robustness of the findings. The complexity and variability of large-scale production systems may influence the reproducibility of the observed effects. In industrial Baijiu fermentation, factors such as larger fermentation volumes, heterogeneous temperature and moisture distributions, and more complex microbial interactions may affect fermentation performance and flavor formation. Therefore, further pilot-scale and industrial-scale studies are required to evaluate the practical applicability and stability of the enriched wheat bran Qu under commercial production conditions.

Secondly, although shifts in bacterial community composition were observed following inoculation, the interactions between the introduced strain and the native microbiota were not fully elucidated. It remains unclear whether *B. cereus* B12 directly interacted with native microorganisms, altered the fermentation environment, or indirectly promoted the enrichment of lactic acid-related genera such as *Levilactobacillus*. In addition, 16S rRNA gene sequencing mainly provides taxonomic information and cannot directly reveal microbial functional activities or specific metabolic pathways. Therefore, future studies integrating microbial interaction analysis, metagenomics, high-throughput sequencing, and metabolomics are needed to clarify microbial succession, functional metabolic pathways, and flavor-related regulation during Baijiu fermentation [55,56,57]. Moreover, water activity was not measured during optimization of moisture content in the present study. As water activity is closely associated with microbial growth and metabolic activity during solid-state fermentation, future studies incorporating water activity analysis may provide further insight into the relationship between moisture conditions and microbial regulation during enriched wheat bran *Qu* preparation. Furthermore, genus-level bacterial community analysis of the enriched wheat bran *Qu* itself was not performed in the present study. Such analysis would provide additional information on the microbial composition of the starter culture and help clarify its role in regulating microbial succession during Baijiu fermentation. Future studies should include microbial community profiling of enriched wheat bran *Qu* to further strengthen the understanding of its functional contribution.

Thirdly, sensory evaluation was not conducted in the present study. Although the increased L-lactic acid proportion and enhanced ester formation suggest potential effects on flavor development, whether these chemical changes lead to improved sensory quality or consumer preference remains to be confirmed. Future studies should include trained sensory panels and consumer preference tests to evaluate the sensory impact of enriched wheat bran Qu on the final Baijiu product.

## 5. Conclusions

In this study, an enriched wheat bran *Qu* containing the L-lactic acid-producing strain *B. cereus* B12 was developed and applied to light-flavor Baijiu fermentation. The preparation conditions of the enriched wheat bran *Qu* were optimized using single-factor experiments and orthogonal design, and the optimal parameters were determined as an inoculation level of 3%, moisture content of 55%, cultivation temperature of 37 °C, and cultivation time of 60 h.

The addition of enriched wheat bran *Qu* influenced the fermentation characteristics of light-flavor Baijiu, including changes in physicochemical parameters and carbohydrate metabolism during fermentation. More importantly, the inoculated fermentation system showed increased production of lactic acid, particularly L-lactic acid, resulting in a higher proportion of the L-lactic acid isomer.

In addition, the concentrations of key ester compounds, including ethyl lactate and ethyl acetate, were significantly increased in the light-flavor Baijiu produced with enriched wheat bran *Qu*. Microbial community analysis further indicated that the inoculation altered the bacterial community structure of fermented grains, with increased abundance of several lactic acid-related genera.

Overall, these findings demonstrate that enriched wheat bran *Qu* containing *B. cereus* B12 can regulate lactic acid metabolism and promote ester formation during light-flavor Baijiu fermentation. This strategy provides a potential approach for modulating L-lactic acid composition and improving flavor formation in Baijiu production. Future studies should further evaluate the industrial applicability and long-term stability of enriched wheat bran *Qu* under commercial fermentation conditions. In addition, integrated sensory evaluation and multi-omics approaches are needed to better elucidate the relationships among microbial regulation, lactic acid metabolism, ester formation, and flavor quality during Baijiu fermentation.

## Figures and Tables

**Table 1:** Factors and levels of the L_9_(3^4^) orthogonal experimental design for enriched wheat bran *Qu* preparation.

Level	A: Inoculation (%)	B: Cultivation Time (h)	C: Moisture Content (%)	D: Cultivation Temperature (°C)
1	1	48	50	35
2	3	60	55	37
3	5	72	60	39

**Table 2:** Results of the L9 (3^4^) orthogonal design.

No.	A: Inoculation Level (%)	B: Cultivation Time (h)	C: Moisture Content (%)	D: Cultivation Temperature (°C)	Microbial Biomass (10^8^ CFU/g)
1	1	48	50	35	33.0
2	1	60	55	37	112.3
3	1	72	60	39	4.7
4	3	48	60	37	68.0
5	3	60	50	39	60.3
6	3	72	55	35	110.3
7	5	48	55	39	55.3
8	5	60	50	35	72.0
9	5	72	60	37	87.0

**Table 3:** α-diversity indices of bacterial communities in fermented grains.

	ACE	Chao 1	Shannon	Coverage (%)
F1	122	121.71	3.03	99.97
F2	172	172.00	3.47	99.99

F1: fermented grains without enriched wheat bran *Qu*. F2: fermented grains with enriched wheat bran *Qu.*

## Data Availability

The original contributions presented in this study are included in the article/Supplementary Materials. Further inquiries can be directed to the corresponding authors.

## Supplementary Materials

The following supporting information can be downloaded at: https://www.mdpi.com/article/10.3390/foods15132290/s1, Table S1: Range analysis of orthogonal design; Figure S1: Changes in D-lactic acid concentration during light-flavor Baijiu fermentation.

## References

[1] Zheng X.-W., Han B.-Z. (2016). Baijiu (白酒), Chinese liquor: History, classification and manufacture. J. Ethn. Foods.

[2] Wu J., Li A., Huang M., Chen H., Sun J., Sun B. (2015). Discovery and Research of Propyl Lactate from 30 Kinds of Chinese Liquors. J. Chin. Inst. Food Sci. Technol..

[3] Tu W., Cao X., Cheng J., Li L., Zhang T., Wu Q., Xiang P., Shen C., Li Q. (2022). Chinese Baijiu: The Perfect Works of Microorganisms. Front. Microbiol..

[4] Gao W., Fan W., Xu Y. (2014). Characterization of the Key Odorants in Light Aroma Type Chinese Liquor by Gas Chromatography–Olfactometry, Quantitative Measurements, Aroma Recombination, and Omission Studies. J. Agric. Food Chem..

[5] Pang X.-N., Chen C., Huang X.-N., Yan Y.-Z., Chen J.-Y., Han B.-Z. (2021). Influence of indigenous lactic acid bacteria on the volatile flavor profile of light-flavor Baijiu. LWT.

[6] Xu H., Qiu S., Dai Y., Wu Y., Zeng X. (2022). Distribution and Quantification of Lactic Acid Enantiomers in Baijiu. Foods.

[7] Zhou Y., Hua J. (2025). Regulation and Mechanisms of L-Lactic Acid and D-Lactic Acid Production in Baijiu Brewing: Insights for Flavor Optimization and Industrial Application. Fermentation.

[8] Nanjo Y., Yano T., Hayashi R., Yao T. (2006). Optically specific detection of D- and L-lactic acids by a flow-injection dual biosensor system with on-line microdialysis sampling. Anal. Sci..

[9] Jiang F., Zhao Z., Nie Y., Li Q., Liu S., Wang H. (2019). Chiral separation and determination of L-and D-lactic acid in Baijiu by high-performance liquid chromatography. Brew. Sci. Technol..

[10] Zhou C., Zheng F., Li H., Wu J., Huang M., Sun B. (2019). Research progress on flavor compounds of Baijiu Daqu. China Brew..

[11] Bai Y., Miao Z., Yan R., Wang X., Cheng Z., Yang J., Wang B., Sun J., Li Z., Zhang Y. (2024). *Daqu* regulates the balance of saccharification and alcoholic fermentation to promote Chinese baijiu fermentation. Food Biosci..

[12] Huang Y., Yi Z., Jin Y., Zhao Y., He K., Liu D., Zhao D., He H., Luo H., Zhang W. (2017). New microbial resource: Microbial diversity, function and dynamics in Chinese liquor starter. Sci. Rep..

[13] Deng H., Huang H., Yang R., Yu K., Liao R., Ma Y. (2025). Effects of Wheat Composition on the Physicochemical and Volatile Components of *Daqu* (A Primary Starter for Chinese Baijiu Fermentation). Foods.

[14] Zhang R., Kang R., Tang D. (2022). Metagenomics-based insights into the microbial community profiling and flavor development potentiality of *baijiu Daqu* and *huangjiu* wheat Qu. Food Res. Int..

[15] Ren Z., Liu L., Tang T., Huang K., Huang Z. (2025). Effectively increase the L(+)-isomer proportion of ethyl lactate in Baijiu by isolating and applying L(+)-lactic acid-producing bacteria. Food Biosci..

[16] Luo B. (2021). Bacillus Applications in Xiaoqu Liquor Brewing.

[17] Zheng Z., Wei C., Deng J., Huang Z., Zhong J., Ren Z. (2021). Study on the production of Fuqu and the characteristics of enzyme about a cellulase producing Bacillus subtilis. Food Mach..

[18] Qiu Y., Wang Y., Zhang W., Sun Q., Luo A. (2023). Strain screening of high-yield tetramethylpyrazine and optimization of its Fuqu process technology. China Brew..

[19] Liu K., Liu Y., Chen N., Ding X., Zhang Z., Liu W. (2023). Study on identification of Bacillus sp. A-1 and its antipathogenic and plant growth-promoting capability. J. Shaanxi Univ. Sci. Technol..

[20] Yan J. (2024). Application of Auxiliary Materials in the Brewing Process of Aromatic *Daqu* Liquor. Liquor Mak..

[21] (2016). National Food Safety Standard—Determination of Moisture Content in Foods.

[22] Pan Q., Zhou Y., Wu M., Hu T., Liu P. (2025). Study on the Influence of Moisture and Acidity of Fermented Grains on the Production of Jiangxiang Baijiu. Yunnan Chem. Technol..

[23] (2016). National Food Safety Standard—Determination of Reducing Sugar in Foods.

[24] (2023). National Food Safety Standard—Determination of Starch in Foods.

[25] (2022). Method of Analysis for Baijiu.

[26] Gibbons W.R., Westby C.A. (1986). Effects of Inoculum Size on Solid-Phase Fermentation of Fodder Beets for Fuel Ethanol Production. Appl. Environ. Microbiol..

[27] Krishna C., Nokes S.E. (2001). Influence of Inoculum Size on Phytase Production and Growth in Solid–State Fermentation by *Aspergillus niger*. Trans. ASAE.

[28] Naveena B.J., Vishnu C., Altaf M., Reddy G. (2003). Wheat bran an inexpensive substrate for production of Lactic acid in solid state fermentation by *Lactobacillus amylophilus* GV6—Optimization of fermentation conditions. J. Sci. Ind. Res..

[29] Sridhar M., Chandrashekaran M. (2011). Solid State Fermentation (SSF) of some Fishery and Agro-Industrial Wastes for Animal Feed Production. Adv. Appl. Res..

[30] Yunus F.-u.-N., Nadeem M., Rashid F. (2015). Single-cell protein production through microbial conversion of lignocellulosic residue (wheat bran) for animal feed. J. Inst. Brew..

[31] Zhang Y., Zhou J., Zhang N., Zhao L., Wu W., Zhang L., Zhou F. (2022). Process Optimization for Production of Ferulic Acid and Pentosans from Wheat Brans by Solid-State Fermentation and Evaluation of Their Antioxidant Activities. ACS Food Sci. Technol..

[32] Subhosh Chandra M.G., Rajasekhar Reddy B. (2013). Exoglucanase production by *Aspergillus niger* grown on wheat bran. Ann. Microbiol..

[33] Ridder E.R., Nokes S.E., Knutson B.L. (1998). Optimization of Solid-State Fermentation Parameters for the Production of Xylanase by *Trichoderma longibrachiatum* on Wheat Bran. Trans. ASAE.

[34] Wang H., Sun C., Yang S., Ruan Y., Lyu L., Guo X., Wu X., Chen Y. (2023). Exploring the impact of initial moisture content on microbial community and flavor generation in Xiaoqu baijiu fermentation. Food Chem. X.

[35] Zhang Z., Wang Y., Yao Y., Li Y., Xu X., Hou Q., Hu X., Mei X., Guo Z. (2025). Microbial and flavor dynamics of medium-high temperature Daqu: Regional influences and implications for *Daqu* quality optimization. Food Res. Int..

[36] Hao Q., Shi Y., Yang Z., Lin C., Wen E. (2025). Study on the Mechanism of Temperature Rise in Koji Room Fermentation based on the Influence of Microbial Metabolism. Int. J. Biol. Life Sci..

[37] Xiao C., Lu Z.-M., Zhang X.-J., Wang S.-T., Ao L., Shen C.-H., Shi J.-S., Xu Z.-H. (2017). Bio-Heat Is a Key Environmental Driver Shaping the Microbial Community of Medium-Temperature Daqu. Appl. Environ. Microbiol..

[38] Yuan H., Zheng J., Ding L., Wang H., Jiang Q., Zhang C., Xie T., Nan G., Li L., Lou K. (2025). Temperature-Driven Divergence in Microbial Consortia and Physicochemical Functionality: A Comparative Study of High- and Medium-Temperature Daqu. Microorganisms.

[39] Acharya A., Subedi S. (2025). Efficient Extraction and Optimization of Amylase and Protease from *Bacillus* Species: A Comprehensive Study. J. Appl. Res. Plant Sci..

[40] Acharya A., Subedi S. (2025). Extraction, Isolation, Purification and Optimization of Amylase and Protease Enzymes Isolated from *Bacillus* species. bioRxiv.

[41] Wang B., Wu Q., Xu Y., Sun B. (2020). Synergistic Effect of Multiple Saccharifying Enzymes on Alcoholic Fermentation for Chinese Baijiu Production. Appl. Environ. Microbiol..

[42] Wang X., Wang B., Sun Z., Tan W., Zheng J., Zhu W. (2022). Effects of modernized fermentation on the microbial community succession and ethyl lactate metabolism in Chinese baijiu fermentation. Food Res. Int..

[43] He G., Huang J., Zhou R., Wu C., Jin Y. (2019). Effect of Fortified *Daqu* on the Microbial Community and Flavor in Chinese Strong-Flavor Liquor Brewing Process. Front. Microbiol..

[44] Xu P., Wu Y., Tian L., Liu Y., Qiu X., Yu J., Liu Q., Shang H., Xiang S., Guan T. (2025). Application of fortified *Daqu* to optimize the core fermentation microbial flora and improve the ester profile of Nongxiangxingbaijiu. LWT.

[45] Cui Y., Wang M., Zheng Y., Miao K., Qu X. (2021). The Carbohydrate Metabolism of *Lactiplantibacillus plantarum*. Int. J. Mol. Sci..

[46] Yan C., Chen X., Liu Q., Xu T., Zhang Q., Jin X., Liao B., Chen X., Li X. (2025). Effects of *Lactiplantibacillus plantarum* on Metabolites and Flavors in Synthetic Microbiota During Baijiu Fermentation. Foods.

[47] Huang X., Fan Y., Meng J., Sun S., Wang X., Chen J., Han B.-Z. (2021). Laboratory-scale fermentation and multidimensional screening of lactic acid bacteria from Daqu. Food Biosci..

[48] Cui Y., Märtlbauer E., Dietrich R., Luo H., Ding S., Zhu K. (2019). Multifaceted toxin profile, an approach toward a better understanding of probiotic *Bacillus cereus*. Crit. Rev. Toxicol..

[49] Fabián J.C.P., Contreras A.K.Á., Bonifacio I.N., Robles M.F.H., Quiñones C.R.V., Ramírez E.I.Q., Salinas C.V. (2025). Analysis of the Pangenome of *Bacillus cereus* and Its Relevance in Food Safety. Agric. Food.

[50] Lapidus A., Goltsman E., Auger S., Galleron N., Segurens B.A., Dossat C., Land M., Broussolle V., Brillard J., Guinebretière M. (2008). Extending the *Bacillus cereus* group genomics to putative food-borne pathogens of different toxicity. Chem.-Biol. Interact..

[51] Wang Z., Wang C., Shen C., Wang S., Mao J., Li Z., Gänzle M., Mao J. (2020). Microbiota stratification and succession of amylase-producing *Bacillus* in traditional Chinese Jiuqu (fermentation starters). J. Sci. Food Agric..

[52] Li Z., Chen L., Bai Z., Wang D., Gao L., Hui B. (2015). Cultivable bacterial diversity and amylase production in two typical light-flavor Daqus of Chinese spirits. Front. Life Sci..

[53] Williams L.D., Burdock G., Jiménez G., Castillo M. (2009). Literature review on the safety of Toyocerin, a non-toxigenic and non-pathogenic *Bacillus cereus* var. toyoi preparation. Regul. Toxicol. Pharmacol..

[54] Takahashi M., Kita Y., Minakami R., Mukai N. (2021). The growth characteristics of *Bacillus cereus* in sake and during its manufacture. J. Food Prot..

[55] Li J., Guo Y., Yang Y., Li S., Xu T., Zeng R., Wang S., Shen C., Xu Z., Zuo Y. (2025). Multi-Omics Insights into Microbial Community Dynamics and Functional Shifts During Double-Round Bottom Fermentation of Strong-Flavor Baijiu. Foods.

[56] Wang Y., Quan S., Zhao Y., Xia Y., Zhang R., Ran M., Wu Z., Zhang W. (2023). The active synergetic microbiota with *Aspergillus* as the core dominates the metabolic network of ester synthesis in medium-high temperature Daqu. Food Microbiol..

[57] Shang S., Qing Y., Zhang D. (2025). Multi-omics Technology-Based Flavor Formation Mechanisms and Intelligent Quality Control Research in Strong-Flavor Baijiu. Int. J. Biol. Life Sci..

