# Supplementary material for: L-Lactic Acid-Enriched Wheat Bran Qu Containing Bacillus cereus Regulates L-Lactic Acid and Ester Formation in Light-Flavor Baijiu Fermentation
Source: Foods. 2026 Jun 26;15(13):2290. doi: 10.3390/foods15132290 (PMC13362162; doi:10.3390/foods15132290)
Supplement: Supplementary file 1 [file foods-15-02290-s001.zip › foods-4372968-supplementary.pdf]

Supplementary Materials:

Table S1. Range analysis of orthogonal design.

Factor	K1	K2	K3	R
A	150.1	238.7	214.3	88.6
B	156.3	244.7	202.1	88.3
C	180.3	278.0	144.8	133.2
D	215.3	267.3	120.4	146.9

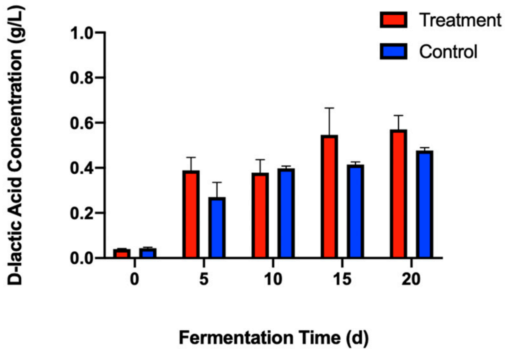

Figure S1. Changes in D-lactic acid concentration during light-flavor Baijiu fermentation.
